# Occurrence and Risk Assessment of Polybrominated Diphenyl Ethers in Surface Water and Sediment of Nahoon River Estuary, South Africa

**DOI:** 10.3390/molecules27030832

**Published:** 2022-01-27

**Authors:** Chinemerem Ruth Ohoro, Abiodun Olagoke Adeniji, Lucy Semerjian, Anthony Ifeanyi Okoh, Omobola Oluranti Okoh

**Affiliations:** 1 SAMRC Microbial Water Quality Monitoring Centre, University of Fort Hare, Alice 5700, South Africa; adenijigoke@gmail.com (A.O.A.); aokoh@ufh.ac.za (A.I.O.); ookoh@ufh.ac.za (O.O.O.); 2 Department of Pure and Applied Chemistry, University of Fort Hare, Alice 5700, South Africa; 3 Department of Chemistry and Chemical Technology, National University of Lesotho, Roma P.O. Box 180, Lesotho; 4 Department of Environmental Health Sciences, College of Health Sciences, University of Sharjah, Sharjah P.O. Box 27272, United Arab Emirates; lsemerjian@sharjah.ac.ae; 5 Applied and Environmental Microbiology Research Group, Department of Biochemistry and Microbiology, University of Fort Hare, Alice 5700, South Africa

**Keywords:** polybrominated diphenyl ethers, risk, physicochemical properties, contamination source, estuary

## Abstract

The concentrations, potential sources, and compositional profile of PBDEs in the surface water and sediment of Nahoon Estuary, East London, South Africa, were investigated with solid-phase extraction and ultra-sonication, respectively, followed by gas-chromatography-electron capture detection. The seasonal range of the contaminants’ concentrations in water and sediment samples in spring season were ∑PBDE 329 ± 48.3 ng/L (25.32–785 ng/L) and ∑PBDE 4.19 ± 0.35 ng/g dw (1.91–6.57 ng/g), but ∑PBDE 62.1 ± 1.50 ng/L (30.1–110 ng/L) and ∑PBDE 65.4 ± 15.9 ng/g dw (1.98–235 ng/g) in summer, respectively. NH1 (first sampling point) was the most contaminated site with PBDE in the Estuary. The potential source of pollution is attributed to the stormwater runoff from a creek emptying directly into the Estuary. This study’s dominant PBDE congener is BDE-17, ranging from below detection limit to 247 ng/L and 0.14–32.1 ng/g in water and sediment samples, respectively. Most detected at all the sites were BDE-17, 47, 66, and 100. Most BDE-153 and 183 are found in sediment in agreement with the fact that higher brominated congeners of PBDE adsorb to solid materials. There was no correlation between the congeners and organic carbon and organic matter. However, the human health risk assessment conducted revealed that the PBDE concentration detected in the estuary poses a low eco-toxicological risk. Nevertheless, constant monitoring should be ensured to see that the river remains safe for the users, as it serves as a form of recreation to the public and a catchment to some neighbourhoods.

## 1. Introduction

Polybrominated diphenyl ethers (PBDEs) are utilized broadly as brominated flame retardants (BFRs) in various materials (such as plastics, electronics, and building materials). They have been listed as Persistent Organic Pollutants (POPs) in the Stockholm Convention in 2009 and 2017 [1], and are resistant to environmental degradation. Lower brominated congeners like tetra-, penta- and hexa- exhibit a high affinity for lipids and are accumulated in the bodies of animals and humans [2]. They are ubiquitous and toxic in the environment and have constituted a pronounced hazard to human health and ecosystems [1]. Evidence shows that tetra- and penta-BDEs are more toxic and bioaccumulative than octa and deca-congeners [2,3,4,5]; and consequently, PBDEs have been linked with thyroid hormone imbalance, tumors, and neurodevelopmental toxicity in children and young adults [2]. There has been global contamination of PBDEs due to the slow removal of PBDEs as they show environmental persistence [2]. Human beings may be exposed to PBDEs by oral intake of contaminated dust, or food, dermal contact with soil or dust, and inhalation of contaminated air [6,7,8].

Contamination of the environment with PBDEs has been reported in several studies in South Africa [9,10,11,12,13]. South Africa is faced with water scarcity, and there are growing demands on this resource with population increase and expansion of the economy. The country must take urgent steps to protect the water quality while providing the wide-ranging necessities for water [14,15]. Materials comprising PBDEs are not given appropriate treatment (incinerated) before discarding into the landfill sites in South Africa, unlike other developed countries like Japan, resulting in high concentration in South Africa, thereby exposing humans to endocrine-disrupting compounds (EDCs) [16]. There is no documented information concerning production volume, distribution, and use of PBDEs in and around Africa, including South Africa [11,17], nor its consumption [17]. There is an assumption that products containing these chemicals are probably imported into South Africa from time to time [16]. Therefore, the trend observed with this chemical could be due to their consumption form or products that contain them [18]. Sources of brominated fire retardants (BFRs) into the South African environment are still vague. However, they may enter the environment during the manufacturing and use of BFR-containing products, disposal, and from varying non-point sources such as atmospheric deposition, urban and agricultural runoffs [19], and the plastic industry which is the largest consumer of BFR [20]. The large metallurgical and chemical industries, smelting and mining companies, and a fast-growing petrochemical-based synthetic sector may also boost the use of BFRs in South Africa [21].

However, there are no existing national laws in South Africa to regulate the production and use of these chemicals. Consequently, the potentially harmful impact would occur if not properly managed [19].

There is a scarcity of reports on PBDEs in the rivers of South Africa, as many reports were on leachates [16,22], sediments [11,19], and fish [12]. Overflow valves release materials into the river during high rainfall periods [23,24,25]. Several estuaries in the Eastern Cape Province, South Africa, such as Sundays and Swartkops, are contaminated with PBDEs, as reported [12,26]. Some other pollution activities have been documented in Nahoon Estuary [15]. Limited irrigation takes place in the horseshoe valley from Nahoon [27], and nitrogen and phosphorus in the system is attributed to pollution source within Nahoon Estuary [28]. Nahoon Estuary is generally prone to regular floods and droughts. The tidal creek at Nahoon Estuary is narrow and small compared to other creeks in South Africa [29]. Many unnamed tributaries enter the Nahoon Dam; for instance, Nqgkana and Kwetyana tributaries enter the Nahoon Dam from the north, and the Rwantsa tributary joins from the west. Furthermore, many small farm dams exist as tributaries of Nahoon River [30].

Final effluents from wastewater treatment plants discharged at Bats Cave into the Indian Ocean, in the middle of Eastern beach and Nahoon, and by the East Bank Reclamation Works, East London, South Africa, can cause the effluents to disperse onto both shores by wave action [16,29,31]. A report also showed that the sewage from municipal wastewater occasionally spilled into Nahoon River [23,24]. This study aims to (1) evaluate the occurrence of PBDEs in the surface water and sediments of the Nahoon River estuary and (2) evaluate the risk associated with PBDEs to human health.

## 2. Materials and Method

### 2.1. Study Area

**Figure 1:**
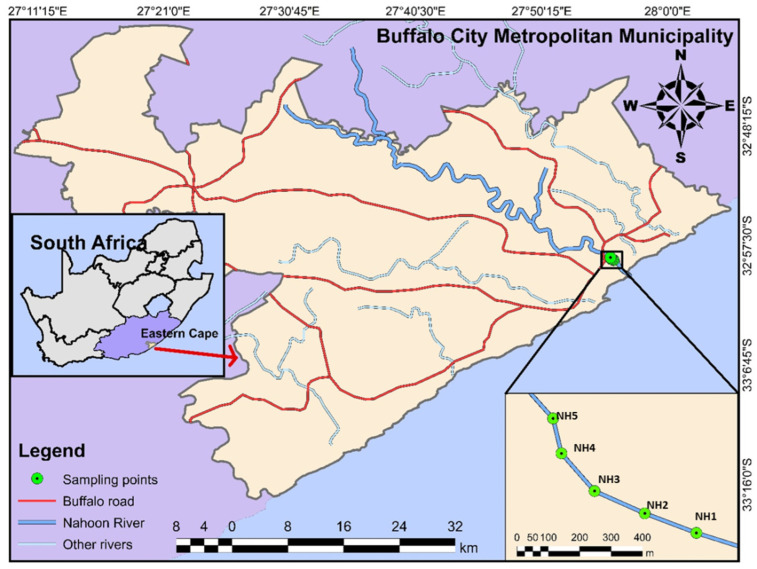
Location map of the study location within South Africa and the five sampling points (NH1, NH2, NH3, NH4, NH5) within the Nahoon River estuary.

The Nahoon Estuary (32°59′ S, 27°57′ E), widely used for weighty recreational boating [32], is located in the warm temperate biogeographic area of South Africa between the Beacon Bay and the East London environs of Nahoon in the Eastern Cape. This perpetually open Estuary is moderately short, and measures 5 km from the mouth through the Abbotsford Bridge, restraining the tidal effect. The Nahoon Estuary was classified as fair in health rank, with adequate loss of habitat and high pollution density, and was apportioned the Ecological Category C [28]. The map of the study site and the sampling coordinates are shown respectively in Table 1 and Figure 1.

### 2.2. Sampling and Sample Pretreatment

All glassware and sampling equipment were soaked overnight with acid(potassium chromate), washed with liquid detergent, and rinsed successively with deionized water and acetone (analytical grade); glasswares were thereafter heated at 105 °C for 4 h before use. Five samples per season for each of water samples were collected in triplicates (altogether 30 water samples), respectively in October and December 2020. No sampling was done in both autumn (March to May) and winter (June to August) seasons due to the global COVID-19 pandemic and logistic reasons during this study. Surface water samples (within 15 cm depth) from the Nahoon River Estuary were collected using a metal bucket sampler into 1 L amber glass bottles, which were twice washed with the sample water before sample collection to avoid cross-contamination. After being transported to the laboratory in iceboxes, the samples were filtered through Whatman No. 1 filter paper (140 μm) within 12 h and extracted immediately. Physicochemical parameters of water were measured on-site using Hanna multi parameters (HI929829) for pH, temperature, total dissolved solids, electrical conductivity, salinity, resistivity, oxidation-reduction potential, and turbidity, HACH (DR900) for total suspended solids, and HACH portable meter (HQ400) for Dissolved oxygen.

Surface sediment samples (about 5 cm depth) were collected with a Van Veen grab sampler at Nahoon River Estuary in triplicate (29 samples altogether). No sediment sample was collected at location NH5 in the spring season (September to November) because of the sampling point’s inaccessibility on that day. The collected samples were separately enfolded in pre-cleaned aluminum foil, conveyed to the laboratory in a cooler box containing ice packs at 4 °C. Pieces of debris and large stones were removed in the laboratory; sediment samples were air-dried in a dark room, blended, and homogenized thoroughly with mortar and pestle, and sieved independently with 250 µm mesh sieves and extracted afterward (an aliquot of the wet sample was reserved for moisture content and an aliquot each was also used for the measurement of organic carbon and organic matter). Organic matter (OM) and organic carbon (OC) of the sediment were determined using the loss on ignition method [33].

### 2.3. Extraction, Purification, and Instrumental Analysis

Water extraction: Extraction was modified and carried out according to the work previously reported [34]. Strata™ C18–E (55 μm, 70 A, 500 mg, 6 mL) solid-phase extraction cartridge from Phenomenex (Torrance, CA, USA) were conditioned with ethyl acetate (6 mL) and distilled water (6 mL) at 1 mL min^−1^ flow rate. Thereafter, 500 mL of the filtered water sample was spiked with surrogate standard (PCB 209, procured from Wellington Laboratories, Ontario, Canada) and extracted through the conditioned cartridge at a flow rate of 10 mL min^−1^_,_ after which the column was dried for 3 hrs, and then the analyte was eluted with 2 × 2.5 mL of ethyl acetate. The extract was evaporated to dryness under a gentle stream of nitrogen and reconstituted with acetonitrile (1 mL) before gas chromatography-micro electron capture detection (GC-µECD) analysis.

Sediment extraction: Sediment extraction was done based on a modified ultrasonically assisted extraction method reported in the literature [22], and analytes were measured with a gas chromatography-electron capture detector (GC-µECD). Approximately 10 g of sample was weighed into a clean amber bottle for ultrasonication (100 mL). Afterward, each of the samples was spiked with surrogate standard (PCB-209) and allowed to soak and equilibrate for 1 h. The samples were allowed to soak overnight afterward with 20 mL acetone: n-hexane (1:2, *v/v*) in an airtight amber bottle (100 mL), followed by extraction at 45 °C for 30 min in an ultrasonicator (LASEC Cape Town, South Africa Pty Ltd., single frequency 40 Hz, 6 L). After sonication, the sediment sample with extract was left to cool and settle for 60 min at 0 °C before the extract was drawn out of the vial using a Pasteur pipette into another bottle. The extracts were centrifuged at 5000 rpm for 5 min. Extraction was done recurrently for two extra times on every single sample with fresh solvents, and the extracts (about 60 mL) were collected. The crude extracts volume containing PBDEs was reduced by rotary evaporator to 1 mL with a Büchi Rotavapor R-210 (vapor: 40 °C, bath temperature: 40 °C, and cooling water temperature: 20 °C). The glass chromatographic column (10 mm × 30 cm) used for cleanup was packed with glass wool, silica gel (1 g; 100–200 mesh), copper powder (2 g; <63 μm) in layers from the bottom with and topped up with 0.5 g Na_2_SO_4_ (anhydrous), separating each layer with glass wool to enhance cleaning. The packed column was pre-saturated with DCM (8 mL), and then n-hexane (8 mL). Approximately 1 mL of the sample extract was put into the column and allowed to run. The adsorbed analyte was then eluted with n-hexane (6 mL) at 5 mL min^−1^ flow rate and transferred carefully into pre-cleaned amber glass tubes. Nitrogen gas (N_2_) was fizzed through the eluate and then concentrated to dryness. The Sample was then reconstituted to 1 mL with n-hexane before injection onto the GC-µECD under optimized instrumental conditions.

Instrumental conditions of this analysis and quality assurance have been reported in our recent study [35].

### 2.4. Statistical/Data Analysis

The following statistics: regression analysis, descriptive analysis, distribution percentage, composition pattern of PBDEs in surface water were calculated with Microsoft Excel 2016. Pearson correlation coefficients between individual compounds and the physicochemical parameters and dendrograms were analyzed using SPSS software package. The percentage distribution of PBDEs was calculated across all seasons. Samples below LOD were treated as zero all through the statistical analysis. Concentrations were shown as ng/g dry weight (dw) for sediment samples, and ng/L for water samples.

### 2.5. Risk Assessment

Risk assessment was conducted for water and sediment samples using a model as shown in Equations (1) and (2) [12,36].

#### 2.5.1. Estimated Daily Intake (EDI)

Estimated daily intake for water samples were calculated using Equation (1) below:

EDI = C × IV/BW(1)

where EDI is the estimated daily intake for target PBDEs (ng/kg bw/day), C is the mean seasonal concentration of PBDE in water (ng/L), BW is the bodyweight of 60 kg [37], and IV is the ingestion volume of 2 L [38].

#### 2.5.2. Non-Carcinogenic Risk

Non-carcinogenic risks for water samples were calculated using Equation (2) below:

HQ = EDI/RFD(2)

where HQ is the hazard quotients, and RfD values (100 ng/kg bw/day for BDE-47, and 200 ng/kg bw/day for BDE-100 and 153) are the US EPA values as reported by US EPA [39].

Eco-toxicological risks posed by PBDE in sediment samples were evaluated by employing the assessment as given in Equation (3) [40,41,42].

HQ = C/PNEC(3)

where C is the concentration of PBDEs in sediment, PNEC is the predicted no-effect concentration below which no adverse effect is envisaged. The concentrations of 31 and 9100 ng/g dw were used for penta and octa-BDE, respectively [43,44]. Tri-penta-BDE (17, 47, 66, 100) and hexa-octa-BDE (153, 183) were assumed to be penta and octa-BDEs, respectively, as classified by USEPA [45].

FEQG values and the homologues grouped into tetraBDE (BDE-47), pentaBDE (BDE-100), and hexaBDE (BDE-153) were extracted from Federal Environment Quality Guideline (FEQG) for PBDEs [46].

HQ < 0.1 specifies no ecotoxicological, HQ between 0.1–1 shows low ecotoxicological risk, HQ between 1–10 indicates moderate ecotoxicological risk, and HQ > 10 indicates high ecotoxicological risk [40,41].

## 3. Result and Discussion

### 3.1. Spatial Distribution, Seasonal Variation, and Potential Sources of Pollution

The concentrations range for ∑_5_PBDEs were 25.3–785 ng/L (mean: 329 ± 48.3 ng/L) in spring water samples and 30.1–110 ng/L (∑_5_PBDEs 62.1 ± 1.50 ng/L) in summer water samples across the sites (Table 2). The PBDEs concentrations across the points of collection from the two matrices (water and sediments) were significantly different (*p* < 0.05). This can be attributed to the non-uniformity of the activities at the sampling sites. Locations NH1 and NH2 are closer to the creek, which is a point of discharge to the Estuary, NH3 is characterized by its leisure activities, and road activities at point NH5 under the bridge. This variation probably contributed to differences in the concentrations of PBDE. The PBDEs concentrations in the spring water samples were higher than in the summer samples. This could be attributed to atmospheric wet deposition and also the heavy runoff of foul-smelling stormwater on the sampling day [47,48]. With spring being a rainy (wet) season in South Africa and windy, both wet and dry depositions are possible, especially in the coastal environment. Higher rainfall always supports more wet deposition than dry ones [49,50,51]. The highest contamination was observed in the spring water sample at NH1 with BDE- 17, 47, and 100 detected at 247, 190, and 178 ng/L, respectively (Table S3); suggesting ubiquitous usage of the commercial penta-BDE at the area [52]. This dominance was also reported elsewhere with BDE-17, 47, and 100 at concentrations 2.0, 5.8, and 0.4 ng/L [12]. This heavy pollution is attributed to a heavy fresh discharge observed at the creek (NH1) on sampling day. The water sample concentration of BDE-17 was below the detection limit in NH4 in spring, NH3, NH4, and NH5 in the summer season but relatively higher in NH5 in spring (137 ng/L) (Table S3). This variance could be a result of the absence of microbial degradation as BDE-17 is one of the major products of microbial degradation of high molecular weight PBDEs in the environment [53]. Point NH5 is a rocky steep under the bridge and occupied by some homeless individuals and characterized by vehicular emission and careless garbage dump by the road users [54,55]. The anthropogenic waste could be why there was a higher concentration at this spot [56] than at NH3 and NH4, which are closer to the potential most polluted area of this sampling site.

The ∑_6_PBDEs in spring sediment samples was 1.91–6.57 ng/g (∑_6_PBDEs 4.19 ± 0.35 ng/g dw), and 1.98–235 ng/g (∑_6_PBDEs 65.4 ± 15.9 ng/g dw) in summer sediment samples (Table 2). The sediment concentration of PBDEs was relatively higher in summer than in spring, with higher temperature in spring notwithstanding; contrary to previous studies, which reported no detection of PBDE concentrations of sediments [57], and higher concentration in summer season than spring [58,59]. A similar report was documented on high summer concentration [58]. This could be attributed to atmospheric wet deposition [60] and high turbidity observed in summer, preventing photodegradation [61,62]. Estuarine sediment acts as a vital sink of PBDEs originated by several anthropogenic activities [63]. The lower concentration of sediment in the spring season is likely as a result of the dilution of overlying water as also reported elsewhere [64]. BDE-17 and 66 showed the relatively highest concentrations in all sampling points, suggesting PBDE contaminants from an industry [65,66], except in NH1 which receives discharges from runoff. Nahoon Estuary is in East London, which is economically developed, densely populated, and characterized by its seaport. Consequently, Nahoon Eastuary could be receiving discharges from industrial waste which might have high PBDE concentration. Pollution of Nahoon Estuary with PBDE could also be attributed to improper plastic waste disposal from tourists, as plastics are a major contributor of PBDE to the environment. No sediment sample was collected at NH5 in spring because of the lack of accessibility of the sampling point on the day of sampling. The point with the highest sediment concentration is NH3. This is where recreational activities and swimming take place. The high concentration of PBDEs could be attributed to anthropogenic pollution as this place is sometimes littered with wastes from leisure activities. There is a possibility of NH3 receiving other forms of waste other than from recreational activities and runoff from the creek.

### 3.2. The Contamination Level of PBDEs in This Current Study Compared with Other Countries

The concentration of PBDEs in the water and sediment samples is summarized in (Table S3, supporting information). The concentration ranges from BDL-247 ng/L in the spring season and BDL-70.2 ng/L in summer for water. PBDEs in sediment ranges from 0.16–3.42 ng/g dw in spring and 0.25–112 ng/g dw in summer. It is worthy of note that this high concentration which seems unusual for a River was observed at the point of discharge of runoff freshly discharged from a creek on sampling day. PBDEs in the Estuary could probably come from flame retardants that contain plastic products and polyurethane foams [67]. Comparing the concentration of water samples in this finding with previous studies in South Africa, the concentrations from this study were higher than reports from Jukskei River, Cape town (below detection limit) [9], Sundays River Estuary (2.5–39.1 ng/L), and Swartkops River (2.5–169 ng/L) in Port Elizabeth [12]. The high concentration from Swartkops was not out of place as runoffs from Motherwell and Markman canal were noticeably discharging into the Swartkops River Estuary [12]. Higher concentrations from South Africa was also reported from Diep River (320–485 ng/L) in Cape Town, which also received effluent discharges from wastewater treatment plant [68]. Although, BDE-209 was investigated in Diep River but was below the detection limit in most cases [68]. Furthermore, the values from this study were higher than 18 ng/L (mean) in the Netherlands [69], and 7.87 ng/L in South China [70]. It was observed that the sediment concentrations in this study were lower than bdl-212 ng/g in the U.S.A, which possibly could be from leaching and emission from a nearby refuse dump [67], but higher than 0.5–3 ng/g in the U.S.A [71]. BDE-47 and 100 which are the most bioaccumulative congeners from the commercial pentaBDE mixture among others [67], were the dominant congeners in all the above-mentioned sites. Compared to Federal Environmental Quality Guideline (FEQG), water and sediment values were below the standards except for the pentaBDE which has higher values than the FEQG standards both in spring and summer seasons for water (4.25–178 and 7.36–11.5 ng/L) and sediment samples (0.21–0.41 and 0.26–25.7 ng/g dw) respectively.

### 3.3. Impact of Physicochemical Parameters on CONCENTRATIONs of PBDEs in Nahoon Estuary

The temperature range across the five sampling points in the spring and summer season was 20.7–21.3 °C and 24.5–26.6 °C, respectively. There was no significant difference in the physicochemical properties of samples collected (Table 3) from the different sampling points (*p* > 0.05). Higher concentrations were observed in water in the spring season than in summer. High temperatures and strong sunshine in summer help the transfer of PBDEs via wet/dry deposition, and consequently, promoting the degradation of higher brominated diphenyl ethers to lower brominated diphenyl ethers [72]. Although, there is a paucity of experimental data accessible on properties and temperature dependence of PBDEs. PBDEs are endothermal, so the solubility increased with increased temperature [73]. Although, sediment also recorded higher levels of pollutants in summer. This could be due to other factors like higher turbidity observed in the summer season. The pH affects the chemical processes of the water body and can help measure the degree of the effluent trail in the water body [74]. However, PBDEs’ chemical features are not affected by pH [75]. The pH range across the five sampling points in the spring and summer season was 8.27–8.65 and 8.3–8.94, respectively. The range of pH for both is the same, implying that the influence pH on the concentrations of PBDE detected in this site is negligible. The salinity values were 28.8–34.4 PSU and 33.5–33.8 PSU for the spring and summer seasons, respectively. Salinity showed no effect on the sorption of BDE-47 on plastics as reported [76], but can influence PBDEs’ distribution [77]. An Increase in salinity decreases the solubility of many chemicals, and causes adsorption of organic contaminants to the suspended particles and then deposited on to the sediment [78]. It was observed that an increase in salinity decreased the concentration of PBDE in the surface water of the study site and vice versa. Conductivity is the extent of water’s capability to conduct an electric current. A Rough estimate of conductivity specifies the water’s mineral content and could be significantly higher in polluted water or any that receives a large quantity of urban runoff [74]. It is an indicator of the salinity of water [79]. The electrical conductivity was about 44.4–46.5 mS/cm and 51.0–55.5 mS/cm for both spring and summer. Higher conductivity brings about warmer water [80], and consequently lower concentration due to degradation. From this study, the concentration of PBDEs in water was higher in spring than in summer because the spring season had a lower conductivity value than summer. The oxidation-reduction property (ORP) of water is a characteristic of the state of natural water. An increase in dissolved oxygen (DO) increases ORP [74]. The Value of ORP for this present study ranges from 23.3–70.8 MvORP and 22.7–55.1 MvORP in summer and spring correspondingly. An increase in temperature and salinity brings about a decrease in DO [74]. The DO of this study ranges from 5–5.59 mg/L and 5.88–8.55 mg/L in summer and spring in that order. Areas of high concentration level cause a decrease in dissolved DO [81]. A higher concentration of PBDEs in water samples with lower DO and vice versa was observed for the spring and summer seasons, respectively. Turbidity slows the degradation of PBDEs such as BDE-209 [82]. In this study, it ranged from 3.97–73 and 10.6–26.4 NTU in summer and spring, respectively. Higher molecular congeners of POPs are known to adsorb to particulate matters and subsequently settle down in the sediment [83,84,85], and turbidity could have prevented debromination of PBDEs by obstructing the penetration of light [82,86] Moreover, natural organic matter is known to inhibit degradation of dibromophenyl ether [87]. Organic matter speeds up gas/particle partitioning. The organic matter in this study ranged from 0.30–0.60% and 0.21–0.27% in summer and spring. This could also explain the reason for higher concentration in spring, as the organic matter was lower. The high concentration of suspended solids suggests potential elevation of hydrophobic endocrine disruptors concentration, which is as a result of compounds having high log K_ow_ values linked with the non-settleable solids [88]. However, lower concentration was observed in summer in this study, probably because of high temperature. The range of TSS was 4.0–15.0 mg/L. TSS is inversely related to particulate organic carbon content [74]. Resistivity assesses the lateral spread of salinity [74] and was measured to be 21.0–22.7 and 19.0–20.0 ohm-cm for spring and summer seasons, respectively. The summary of the physicochemical properties of the water collected from the study area is presented in Table 3.

### 3.4. Correlation of PBDEs in Water and Sediment with Physicochemical Properties

The Pearson correlation analysis between PBDEs concentration in water and sediment with the physicochemical parameters was performed (Tables S1 and S2). Correlation values for BDE-17 versus 47 (r = 0.91, *p* < 0.01), BDE-100 (r = 0.79, *p* < 0.01), BDE-183 (r = 0.78, *p* < 0.01), were observed in water samples. All the congeners correlated strongly among themselves except for BDE-153, which showed no correlation with BDE- 17, 47, and 183. The positive correlation effect suggested that the congeners all came from the same source, except BDE-17. The temperature and pH did not show any correlation with the congeners. Correlation values for EC versus BDE-17 (r = −0.87, *p* < 0.01), BDE-47 (−0.77, *p* < 0.01), BDE-100 (−0.77, *p* < 0.01), and BDE-183 (−0.86, *p* < 0.01) were observed. This implies that the congeners decrease with an increase in EC. There was no correlation between EC, TSS, and do with BDE-153. The correlation of EC with all the physicochemical parameters was observed, only pH correlated with EC and resistivity. A negative correlation was observed between salinity and all congeners except BDE-153 with a very poor correlation (r = 0.128), suggesting a “salting out” effect [78]. In sediment samples, BDE-47, 100, 153, and 183 showed a very strong correlation with all except BDE-17. There was no correlation between the congeners with the OM and OC, all the congeners and physicochemical parameters correlated with moisture content. The observed positive correlation may propose a potential impact of physicochemical parameters on the PBDEs concentrations.

### 3.5. Contamination Pattern and Source Apportionment of Pollution in Nahoon Estuary Using a Dendrogram

**Figure 2:**
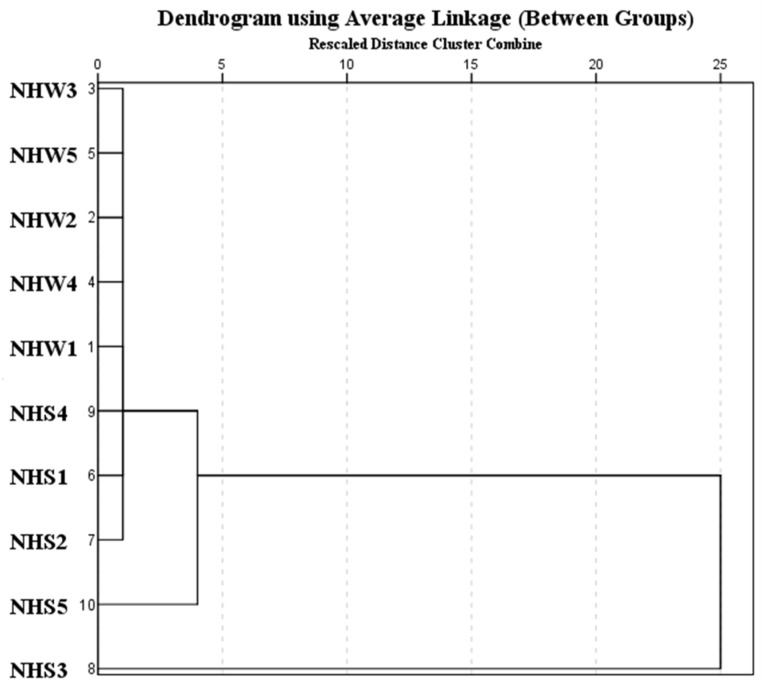
Dendrogram showing the source tracking of the polluted sites.

Dendrogram demonstrating the ranked cluster analysis [89] of PBDEs of water and sediment samples from the 5 sampling points at the Nahoon River Estuary is illustrated (Figure 2). Locations in the same clusters show similar contamination. The study site of PBDEs concentrations was classified with hierarchical cluster analysis by employing the rescaled distance cluster combined with the average linkage method as reported elsewhere [90]. The mean water samples (NHW1, NHW2, NHW3, NHW4, NHW5) and sediment samples (NHS1, NHS2, NHS3, NHS4, NHS5) across the sites were represented. The cluster analysis showed that the area of study is clustered into four major groups based on their mean concentrations in both water and sediment. The first group is NHS3. This is the site with the highest concentration of PBDEs. BDE-183 has the highest concentration of all the congeners on this site. This is possibly an indication of the presence of commercial octa-BDE from a local anthropogenic source [52]. The second cluster comprises NHS5 with the second-highest concentration of PBDEs, having BDE-17 as the highest concentration from this point. The third cluster consists of NHS2, NHS1, and NHS4. These are the sites with the third-highest concentrations of PBDEs. The fourth cluster consists of NHW1, NHW4, NHW2, NHW5, and NHW3, which is a class of clusters with low concentrations.

### 3.6. Compositional Patterns of PBDEs in the Surface Water and Sediments

**Figure 3:**
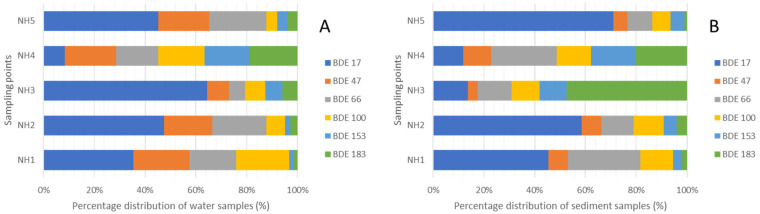
Percentage distribution for (**A**): water and (**B**): sediment samples.

The summary of the percentage distribution of the ∑_6_PBDE is given in Figure 3. In water samples, the dominant congener is BDE-17 (43%), which came majorly from NH1 > NH2 > NH3 > NH4, followed by BDE-47 (27%), and BDE-100 (26%) correspondingly, all suggesting that NH1 and NH2 are the most polluted points which are the closest point to the creek where the runoff from an unknown source is being discharged to the Estuary. The dominant congener in the sediment is BDE-183 (33%), majorly at point NH3, followed by BDE-17 (27%), emanating from NH5 and NH3, BDE-66 (15%), coming from NH3 and NH1, and BDE-100 (11%), coming from NH3, NH5, NH1. All the congeners are detected at NH4 in low concentrations. The highest concentrations of the congeners in sediments were from point NH3, with BDE-183 having the highest concentration (112 ng/g). BDE-183 is an indicator of octa-BDE; therefore, the relatively high concentration is an indication of possible extra contribution from octa-BDE products as similarly reported [64]. Furthermore, lower brominated congeners could be because of debromination or higher congeners or extensive use of products of penta-BDE mixtures as stated earlier.

### 3.7. Ecotoxicological Risk Assessment

The result from the ecotoxicological risk is given (Table 4), which indicates that HQ for water samples shows no risk in the concentrations of PBDEs, while pentaBDE in sediment samples suggests possible low non-cancer risk (0.2). To further evaluate the probable eco-toxicological risk of PBDEs in water and sediment samples in Nahoon River Estuary, the Federal Environment Quality Guidelines (FEQG) standards for PBDEs of Canada [46] were adopted as was also reported elsewhere [91]. The mean concentrations from the two seasons each (Table 5) were compared with BDE-17, 66, and 183 were not included in the homologues in the evaluation of the ecotoxicological risk as they were not given in this guideline. It was observed that hexaBDEs and tetraBDE in both water and sediments pose no risk. However, pentaBDE in both water and sediment samples for the two seasons was above the standard values for FEQG. Although, the concentrations of pentaBDE from this study is higher than the standard values for FEQG; it does not pose threat to water, though a low potential non-cancer risk for sediment is envisaged. Therefore, this calls for special concern to ensure the safety of aquatic lives, tourists, and athletes.

## 4. Conclusions

This present study investigated PBDE (BDE-17, 47, 66, 100, 153, and 183) in water and sediment samples from Nahoon River Estuary using SPE and USE, respectively. Quantification was done with GC-µECD. BDE-17 was the highest detected congener. NH1 appeared to be the highest contaminated location for water samples, while NH3 has the highest concentration in sediment. Spring season gave higher concentration (329 ng/L) than summer in water samples, while sediment samples gave higher concentration in summer season (65.4 ng/g). OM and OC did not correlate with the congeners. The pentaBDE detected in both matrices was higher than the FEQG. However, this concentration is not high enough to cause eco-toxicological risk in water samples, but low eco-toxicological risk is envisaged in sediment samples. This research is therefore essential to Nahoon River Estuary because of its significance in use for recreation. It has shown that even with the efficiency of restraint and prohibition on Nahoon River pollution, PBDE is being detected in the Estuary. Albeit the Estuary’s proper monitoring should be adopted to ensure that the runoff entering does not raise the hazard quotient of the contaminants in the Estuary. It is worth mentioning that the seasonal variations of PBDEs analysed in this study were only those for spring and summer. This may not reflect the whole seasonality of these pollutants in Nahoon Estuary since the South African climate comprises of four seasons (autumn and winter included). As a result, further studies should consider analysing these compounds in environmental matrices throughout the course of four seasons to better understand their seasonal prevalence.

## Figures and Tables

**Table 1:** Sampling sites coordinates and descriptions.

Site Code	Latitude	Longitude	Description
NH1	32°58′36.7″ S	25°55′49.1″ E	Creek
NH2	32°58′34.7″ S	27°55′43.8″ E	Open place
NH3	32°58′32.4″ S	27°55′38.6″ E	Outdoor recreation
NH4	32°58′28.5″ S	27°55′35.1″ E	Open air for recreation
NH5	32°58′24.9″ S	27°55′34.3″ E	Under the bridge

**Table 2:** Mean concentrations of PBDEs in surface water and sediments of Nahoon River estuary in ng/L and ng/g dw, respectively.

	Spring				Summer			
Surface water								
Congener	Mean (n = 5)	Max	Min	DF (100%)	Mean (n = 5)	Max	Min	DF (100%)
BDE 17	140 ± 91.2	247	BDL	80	23.5 ± 31.5	70.2	BDL	40
BDE 47	68.9 ± 76.7	190	4.27	100	7.92 ± 2.80	12.4	5.43	100
BDE 100	44.0 ± 64.4	178	4.25	100	8.95 ± 2.96	11.5	7.36	100
BDE 153	5.88 ± 75.7	6.39	4.97	100	6.59 ± 1.77	8.00	5.82	100
BDE 183	5.79 ± 0.98	5.56	5.41	100	7.02 ± 1.01	8.80	5.13	100
∑PBDE	329 ± 48.3				62.1 ± 1.50			
Sediment								
BDE 17	1.42 ± 1.14	2.77	0.44	100	17.4 ± 12.2	32.0	0.14	100
DE 47	0.27 ± 0.03	0.29	0.24	100	3.14 ± 3.25	8.75	0.28	100
BDE 66	1.47 ± 1.36	3.42	0.27	100	8.88 ± 17.8	31.4	0.29	100
BDE 100	0.20 ± 0.08	0.41	0.21	100	7.25 ± 10.4	25.69	0.26	100
BDE 153	0.19 ± 0.08	0.27	0.08	100	6.00 ± 10.7	25.1	0.63	100
BDE 183	0.56 ± 0.07	0.64	0.47	100	22.7 ± 49.9	112	0.25	100
∑PBDE	4.19 ± 0.35				65.4 ± 15.9			

BDL: below detection limit; DF—frequency of detection; *n* = number of samples.

**Table 3:** Physicochemical properties of surface water and sediment samples.

	Spring		Summer	
Parameters	Mean ± STD	Range	Mean ± STD	Range
Temp. [°C]	21.0 ± 0.28	20.7–21.3	25.9 ± 0.79	24.5–26.6
pH	8.48 ± 0.17	8.27–8.66	8.65 ± 0.22	8.33–8.94
EC [mS/cm]	45.7 ± 1.02	44.4–46.5	51.5 ± 61.0	51.0–51.5
TDS [g/L]	22.9 ± 0.51	22.2–23.3	25.6 ± 80.1	25.5–25.7
Sal. [psu]	29.7 ± 0.74	28.8–34.4	33.6 ± 0.12	33.5–33.8
Turb. [FNU]	17.8 ± 5.97	10.6–26.4	31.4 ± 28.8	3.97–73.0
mVorp	32.2 ± 13.1	22.7–55.1	55.4 ± 19.3	23.2–70.8
RES [Ohm-cm]	21.9 ± 0.60	21.0–22.7	19.6 ± 0.43	19.0–20.0
DO [mg/L]	6.84 ± 1.11	5.88–8.55	5.31 ± 0.26	5.00–5.59
TSS [mg/L]	8.33 ± 3.25	4.00–12.7	9.13 ± 4.21	4.00–15.0
% MC	63.3 ± 1.05	62.5–64.8	30.0 ±3.69	25.0–35.0
% OC	0.25 ± 0.02	0.21–0.27	0.29 ± 0.07	0.17–0.35
% OM	0.42 ± 0.04	0.37–0.47	0.50 ± 0.12	0.30–0.60

Temp—temperature; TDS—total dissolved solid; EC—electrical conductivity; Sal—salinity; mVorp—oxidation-reduction potential; Turb—turbidity; DO—dissolved oxygen; TSS, total suspended solid; MC—moisture content; OC—organic content; OM—organic matter.

**Table 4:** Hazard quotient (HQ) for ecotoxicological risk.

Water			
Congener	Mean (ng/L)	EDI (ng/L)	HQ
BDE 47	38.4	1.28	0.0
BDE 66	36.6	1.22	0.0
BDE 100	26.5	0.88	0.0
BDE 153	6.23	0.21	0.0
Sediment			
Homologue	Mean(ng/g dw)	PNEC (ng/g) *	HQ
PentaBDE	5.48	31	0.2
OctaBDE	14.7	9100	0.0

* PNEC values for water (ng/L) and sediment (ng/g) extracted from literature [42]; EDI = Estimated daily intake; HQ = Hazard quotient.

**Table 5:** Comparison of the PBDEs concentration of the present study with FEQG for both water (ng/L) and sediment (ng/g dw).

			This Study	
Homologue	Congener	FEQG **	Spring	Summer
Water				
TetraBDE	BDE-47	24	4.27–190	5.43–12.4
PentaBDE	BDE-100	0.2	4.25–178	7.36–11.5
HexaBDE	BDE-153	120	4.97–6.39	5.82–8.00
HeptaBDE	BDE-183	17	5.41–3.56	5.13–8.80
Sediment				
TetraBDE	BDE-47	39	0.24–0.29	0.28–8.75
PentaBDE	BDE-100	0.4	0.21–0.41	0.26–25.7
HexaBDE	BDE-153	440	0.08–0.27	0.63–25.1

** FEQG values extracted from the literature [46].

## Data Availability

All the data generated and analysed during the current study in this article are included in this published article.

## Supplementary Materials

The following supporting information can be downloaded. Table S1: Matrix of Pearson correlation among analytes and water quality parameters; Table S2: Matrix of Pearson correlation among sediment parameters; Table S3: Concentrations of PBDEs in surface water and sediments of sampling sites in Nahoon River Estuary in ng/L and ng/g, respectively.
