# Supplementary material for: Occurrence and Risk Assessment of Polybrominated Diphenyl Ethers in Surface Water and Sediment of Nahoon River Estuary, South Africa
Source: Molecules. 2022 Jan 27;27(3):832. doi: 10.3390/molecules27030832 (PMC8839697; doi:10.3390/molecules27030832)
Supplement: Supplementary file 1 [file molecules-27-00832-s001.zip › molecules-1525218-supplementary.pdf]

Occurrence and Risk Assessment of Polybrominated Diphenyl Ethers in Surface Water and Sediment of Nahoon River Estuary, South Africa

Chinemerem Ruth Ohoro [1,2,*], Abiodun Olagoke Adeniji [1,2,3], Lucy Semerjian [4], Anthony Ifeanyi Okoh [1,4,5] and Omobola Oluranti Okoh [1,2]

1 SAMRC Microbial Water Quality Monitoring Centre, University of Fort Hare, Alice 5700, South Africa; adenijigoke@gmail.com (A.O.A.); aokoh@ufh.ac.za (A.I.O.); ookoh@ufh.ac.za (O.O.O.)
2 Department of Pure and Applied Chemistry, University of Fort Hare, Alice 5700, South Africa
3 Department of Chemistry and Chemical Technology, National University of Lesotho, Roma P.O. Box 180, Lesotho
4 Department of Environmental Health Sciences, College of Health Sciences, University of Sharjah, Sharjah P.O. Box 27272, United Arab Emirates; lsemerjian@sharjah.ac.ae
5 Applied and Environmental Microbiology Research Group, Department of Biochemistry and Microbiology, University of Fort Hare, Alice 5700, South Africa
* Correspondence: greatnemerem@yahoo.co.uk

Supplementary Tables

Table S1: Matrix of Pearson correlation among analytes and water quality parameters

	BDE 17	BDE 47	BDE 100	BDE 153	BDE 183	Temp.[°C]	pH	EC[MS/cm]	TDS [g/L]	Sal.[psu]	Turb.FNU	ORP[mV]	RES[Ohm-cm]	DO	TSS
BDE 17	1														
BDE 47	**0.912**	1													
BDE 100	**0.786**	**0.933**	1												
BDE 153	0.287	0.338	**0.474**	1											
BDE 183	**0.78**	**0.816**	**0.731**	-0.22	1										
pH	0.369	0.036	0.017	-0.25	0.348	1									
EC	**-0.87**	**-0.77**	**-0.77**	-0.09	**-0.86**	**-0.64**	1								
TDS	**-0.81**	**-0.86**	**-0.79**	0.127	**-1**	-0.33	**0.883**	1							
Sal	**-0.81**	**-0.86**	**-0.79**	0.128	**-1**	-0.34	**0.883**	1	1						
Turb	**-0.81**	**-0.86**	**-0.79**	0.13	**-1**	-0.34	**0.884**	1	1	1					
Temp	0.215	0.035	0.252	0.285	0.104	**0.73**	**-0.55**	-0.14	-0.15	-0.15	1				
Mvorp	**0.787**	**0.905**	**0.991**	**0.438**	**0.754**	0.13	**-0.83**	**-0.81**	**-0.81**	**-0.81**	0.361	**1**			
RES	**0.449**	**0.529**	**0.716**	**0.037**	**0.704**	**0.424**	**-0.79**	**-0.73**	**-0.73**	**-0.73**	**0.629**	**0.791**	1		
TSS	**-0.5**	-0.39	**-0.53**	-0.05	**-0.58**	-0.77	**0.851**	**0.606**	**0.608**	**0.609**	**-0.864**	**-0.638**	**-0.88**	**1**	
DO	**0.996**	**0.934**	**0.809**	0.342	**0.756**	0.284	**-0.83**	**-0.79**	**-0.79**	**-0.79**	**0.162**	**0.799**	**0.416**	**-0.44**	**1**

Table S2: Matrix of Pearson correlation among sediment parameters

	BDE 17	BDE 47	BDE 66	BDE 100	BDE 153	BDE 183	MC	OC	OM
BDE 17	1								
BDE 47	**0.589**	1							
BDE 66	0.379	**0.964**	**1**						
BDE 100	0.391	**0.972**	**0.988**	**1**					
BDE 153	0.37	**0.952**	**0.966**	**0.991**	**1**				
BDE 183	0.262	**0.921**	**0.963**	**0.985**	**0.993**	1			
MC	**-0.73**	**-0.94**	**-0.836**	**-0.86**	**-0.82**	**-0.77**	1		
OC	0.351	0.396	0.353	0.294	0.171	0.16	**-0.564**	**1**	
OM	0.351	0.396	0.353	0.294	0.171	0.16	**-0.564**	**1**	1

Table S3: Concentrations of PBDEs in surface water and sediments of sampling sites in Nahoon River Estuary in ng/L and ng/g, respectively

	Spring					Summer				
Surface water Congener	NH1	NH2	NH3	NH4	NH5	NH1	NH2	NH3	NH4	NH5
BDE 17	246.96	192.15	121.70	BDL	136.80	70.20	47.43	BDL	BDL	BDL
BDE 47	189.90	89.32	4.94	4.27	56.33	6.52	6.41	12.39	8.87	5.43
BDE 100	178.34	93.95	4.25	4.43	5.69	8.68	9.82	11.53	7.36	7.38
BDE 153	6.39	4.97	5.11	5.59	7.31	7.31	5.82	8.00	5.94	5.86
BDE 183	5.56	5.81	5.72	5.41	6.42	8.80	8.36	5.97	6.83	5.14
Sediment										
BDE 17	1.96	2.77	0.50	0.45	-	13.87	15.35	32.10	0.14	25.47
BDE 47	0.29	0.24	0.29	0.27	-	2.43	2.22	8.75	0.28	2.01
BDE 66	3.42	1.18	0.27	0.99	-	6.44	2.72	31.39	0.29	3.54
BDE 100	0.26	0.27	0.21	0.41	-	4.29	3.44	25.69	0.26	2.57
BDE 153	0.08	0.27	0.16	0.23	-	1.01	1.29	25.12	0.63	1.96
BDE 183	0.54	0.59	0.47	0.64	-	0.25	0.69	111.98	0.37	0.36

BDL: below detection limit
